# Association between social networks and cognitive impairment among older Chinese adults: the mediating effect of depression

**DOI:** 10.3389/fnagi.2024.1495694

**Published:** 2025-01-13

**Authors:** Zhuo Zhang, Ying Bian

**Affiliations:** ^1^School of Health Services Management, Xi'an Medical University, Xi'an, Shaanxi, China; ^2^Institute of Chinese Medical Sciences, State Key Laboratory of Quality Research in Chinese Medicine, University of Macau, Taipa, Macao SAR, China

**Keywords:** social networks, cognitive impairment, depression, mediating effect, older Chinese adults

## Abstract

**Objectives:**

This study aimed to explore the rationality of the social networks-depression-cognitive impairment pathway and to provide recommendations for the development of mild cognitive impairment intervention strategies.

**Methods:**

A cross-sectional survey was conducted in 2021. Sixteen urban communities in Xi 'an, Shaanxi China were selected as sample sites. The cognitive function, social networks and depression were measured by the Montreal Cognitive Assessment (MoCA), the Lubben Social Network Scale-6 (LSNS-6) and the Geriatric Depression Scale-15 (GDS-15), respectively. The generalized linear model was used to analysis the impact of social networks on cognitive impairment, and further analysis the mediating effect of depression.

**Results:**

A total of 745 elderly people aged 60 and above was included in this survey, with an average age of 68.90 ± 6.00 years. The prevalence of cognitive impairment was 18.52%, and the prevalence of cognitive impairment increased with age. According to the generalized linear model, poor social networks (relative network, friend network) was associated with higher risk of cognitive impairment (OR = 2.08, 95% CI: 1.27–3.41), and this association was more significant in women and older adults <70 years of age. Mediation analysis results showed that depression was the mediating path between social networks and cognitive impairment, with the indirect effects accounting for 34.44%.

**Conclusion:**

Social isolation increases the risk of cognitive impairment and depression has a significant mediating effect on the relationship between social isolation and cognitive impairment.

## 1 Introduction

Cognitive impairment (Kuang et al., 2021), as a chronic non-communicable disease, is becoming an important public health problem in China. Cognitive impairment refers broadly to various degrees of cognitive impairment from various causes, ranging from mild cognitive impairment (MCI) to dementia. Dementia is the most severe stage of cognitive impairment and is the leading cause of disability in people over 60 years of age worldwide. To date, there are no effective treatments for these diseases due to the unclear pathogenic mechanisms and failures in clinical trials for drugs developed. Therefore, early intervention before conversion to dementia becomes an important strategy for dementia prevention.

MCI, a potentially transformative state between normal aging and dementia, is recognized as a precursor to Alzheimer's disease (AD) and other types of dementia, providing a unique “window of time” for secondary prevention of dementia. Epidemiological studies have shown that the risk of progression to dementia in MCI patients is 10 times higher than in normal older adults. Some scholars have used models to estimate the influence of a 10% or 25% reduction in risk factors on the prevalence of dementia. Using an estimate of 7.7 million new cases per year, it was concluded that a 10% reduction in risk factor exposure would have resulted in 250,000 (3.3%) fewer new cases per year globally, whereas a 25% reduction in risk factor exposure would have prevented 680,000 (8.8%) new cases per year (Rost et al., 2022). It has been shown that modification of 12 risk factors may prevent or delay up to 40% of dementia (Luttenberger et al., 2012). Since the AD process is irreversible, it is significant to explore possible influencing factors and influencing mechanisms at the MCI stage and to develop appropriate intervention strategies.

Social networks are of particular interest among numerous intervention strategies because improving factors associated with social networks is a relatively simple and feasible way to improve cognitive function. The association of social network as an independent variable with cognitive health outcomes has been partially confirmed (Houtjes et al., 2014). A rich social network has been shown to be a particularly effective intervention for delaying dementia and MCI. Studies have shown that being socially active, even at an advanced age, can delay the onset of dementia by more than a year (Paillard-Borg et al., 2012). There is growing evidence that older adults who have been in a resourceful social network exhibit better cognitive abilities than their socially disengaged peers (Kuiper et al., 2015). However, few studies have elucidated the mechanisms underlying the relationship between social networks and cognitive impairment in older adults. With the increased emphasis on the biopsychosocial model, it is of great relevance whether social networks could play a role in cognitive health through a psychological pathway.

While an association has been found between social networks and MCI, the underlying factors and psychological mechanisms remain largely unexplored. Currently, only a few epidemiological studies have examined the link between social networks and cognitive health. Findings from empirical studies have shown that the quality rather than the quantity of social networks is protective against cognitive decline (Krueger et al., 2009) and that the emotional support appears to be more beneficial than instrumental support (Amieva et al., 2010). It can therefore be hypothesized that depression is a better predictor of MCI than social networks. It is expected that this mechanism is an indirect one, particularly since the outcome of cognitive function is being explained by the perceived rather than directly received quality of social networks. By investigating cognitive function, social networks and depression status of the elderly population in urban communities, this study aimed (1) to investigate the current prevalence of cognitive impairment in older adults in the community, (2) to explore the direct effects of social networks on cognitive function in older adults, (3) to explore the rationale for the Social Network-Depression-Cognitive Impairment pathway, to provide recommendations for the development of MCI intervention strategies, and to provide an important theoretical framework for clinical practice and public health efforts to prevent dementia in older adults. Exploring the mediating pathways between social networks and MCI could help find more effective ways to provide useful information for early detection and prevention of MCI.

## 2 Methods

### 2.1 Data sources and study population

This study was a cross-sectional survey conducted in Xi'an, which was located in the middle of the Guanzhong Plain. Using a stratified sampling method, two districts were first randomly selected from among the districts of Xi'an, two streets were randomly selected in each district, and four communities were randomly selected in each street, resulting in a total of sixteen communities being selected. In each community, 45 older adults were selected with the following inclusion and exclusion criteria.

Inclusion criteria: ≥60 years of age; no intellectual disability, able to understand and answer questions; no major illnesses in the past year. Exclusion criteria: inability to complete the questionnaire independently/assisted; refusal to sign the informed consent form.

The sample size was calculated using the sample size calculation formula of the cross-sectional survey, which was as follows:


$$\begin{array}{l} N=\frac{z^{2}p\left(1-p\right)}{e^{2}} \end{array}$$

With a maximum response distribution rate (*P* = 0.5, e = 0.1p) and 95% confidence level (*z* = 1.96), 385 samples were needed in this study. In addition, considering the stability of the regression model, which usually requires 10–15 times the independent variable, the final sample size of this study should be 140–210. Finally, 745 older adults were surveyed in this study, which meets the sample size requirement.

### 2.2. Social networks, cognitive impairment and depression

Cognitive function was measured using the MoCA (Beijing version). The scale includes cognitive dimensions such as visuospatial ability, naming, attention, language, abstract reasoning, memory, and orientation to time and place. The scale consists of 30 items. The higher the score, the better the neurocognitive function.

Social networks were assessed using LSNS-6. The LSN-6 measures the social isolation of older adults by measuring perceived social support from both family and friends, with each section consisting of three equally weighted items, each consisting of “none,” “1,” “2,” “3–4,” “5–8,” and “9, or more,” with a higher score indicating more social engagement. A total score of less than 12 is considered to have social impairment.

Mental health was measured using GDS-15. The scale has 15 items, each of which is scored as 0 or 1, with a maximum score of 15, with higher scores indicating more pronounced depressive symptoms and ≥5 indicating possible depression.

### 2.3 Covariance

The covariates involved in this study and their grouping included gender (male, female), age (<70, ≥70), marital status (married, others), night shift work (no, yes), living alone (no, yes), smoking status (no, yes), drinking status (no, yes), regular exercise (no, yes), sleep duration (<7, 7–9, ≥9), SES (poor, medium, good). In this study, education level, occupation and income were adopted to reflect the SES of the subjects.

### 2.4. Statistical analysis

The basic characteristics of the study population were presented using descriptive statistics, with continuous variables statistically described using mean (x) ± standard deviation (SD) and categorical variables using frequency (*n*) and composition ratio (%). The factors influencing cognitive impairment were modeled using a generalized linear model. Two models were created, model 1 adjusting nothing and model 2 adjusting covariates. The effects of different social network types (relative network and friend network) on cognition were further analyzed. Principal component analysis was used to construct the SES, and the corresponding weight was shown in Table 1. SES were divided into three groups based on quantile. Social network was the independent variable, cognitive impairment was the outcome variable, and depression was the mediating variable. The commanding *sgmediation2* was employed to conduct mediation analysis, and the calculation was based on “product of coefficients” approach. All statistical procedures were completed using STATA 15.1, and all tests were two-sided, *a* = 0.05.

**Table 1:** Socioeconomic status weights.

**Variable**	**Weight**
Education level	0.410
Occupation	0.696
Income	0.589

## 3 Results

### 3.1 Sociodemographic characteristics of the subjects

**Figure 1:**
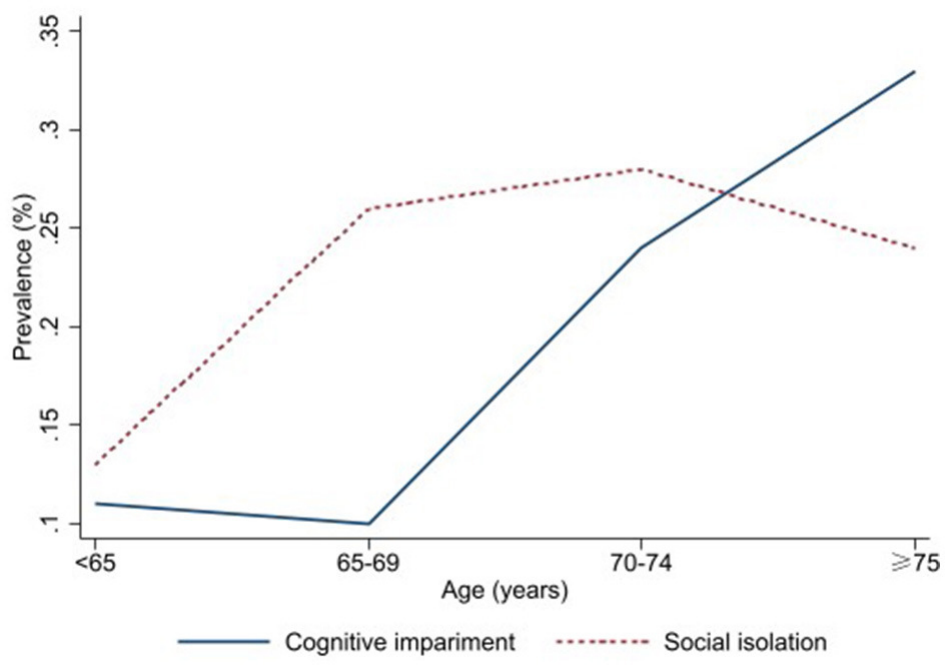
The prevalence of cognitive impairment and social isolation by age.

A total of 745 participants with an average age of 68.90 ± 6.00 years were included in this study, among whom 73.69% were female, 65.10% were married, 10.34% smoked, and 10.74% drank alcohol. Other socio-demographic information was shown in Table 2. The prevalence of cognitive impairment and social isolation in the study subjects was 18.52% and 23.09%, respectively. Figure 1 showed the prevalence of cognitive impairment and social isolation among different age groups. The analysis of factors influencing cognitive impairment showed differences in the prevalence of cognitive impairment by age, living alone, social isolation, alcohol consumption, exercise, sleep duration, and SES groups (Table 2).

**Table 2:** Sociodemographic characteristics of subjects.

		**Cognitive impairment**	
		**No (607)**	**Yes (138)**
**Gender**			
Male	196 (26.31%)	159 (26.19%)	37 (26.81%)
Female	549 (73.69%)	448 (73.81%)	101 (73.19%)
**Age (years)**			
< 70	407 (54.63%)	364 (59.97%)	43 (31.16%)^*^
≥70	338 (45.37%)	243 (40.03%)	95 (68.84%)
**Marital status**			
Married	485 (65.10%)	416 (68.53%)	69 (50.00%)^*^
Others	260 (34.90%)	191 (31.47%)	69 (50.00%)
**Night shift work**			
No	715 (95.97%)	587 (96.71%)	128 (92.75%)^*^
Yes	30 (4.03%)	20 (3.29%)	10 (7.25%)
**Living alone**			
No	629 (84.43%)	525 (86.49%)	104 (75.36%)^*^
Yes	116 (15.57%)	82 (13.51%)	34 (24.64%)
**Social isolation**			
No	573 (76.91%)	498 (82.04%)	75 (54.35%)^*^
Yes	172 (23.09%)	109 (17.96%)	63 (45.65%)
**Depression**			
No	583 (78.26%)	507 (83.53%)	76 (55.07%)^*^
Yes	162 (21.74%)	100 (16.47%)	62 (44.93%)
**Current smoker**			
No	668 (89.66%)	554 (91.27%)	114 (82.61%)^*^
Yes	77 (10.34%)	53 (8.73%)	24 (17.39%)
**Current drinker**			
No	665 (89.26%)	557 (91.76%)	108 (78.26%)^*^
Yes	80 (10.74%)	50 (8.24%)	30 (21.74%)
**Regular exercise**			
No	267 (35.84%)	205 (33.77%)	62 (44.93%)^*^
Yes	478 (64.16%)	402 (66.23%)	76 (55.07%)
**Sleep duration (hours)**			
< 7	298 (40.00%)	224 (36.90%)	74 (53.62%)^*^
7–9	381 (51.14%)	340 (56.01%)	41 (29.71%)
≥9	66 (8.86%)	43 (7.08%)	23 (16.67%)
**SES**			
Poor	251 (33.69%)	180 (29.65%)	71 (51.45%)^*^
Medium	301 (40.40%)	255 (42.01%)	46 (33.33%)
Good	193 (25.91%)	172 (28.34%)	21 (15.22%)

^*^P < 0.05.

### 3.2. The correlation between cognitive impairment and social network

**Figure 2:**
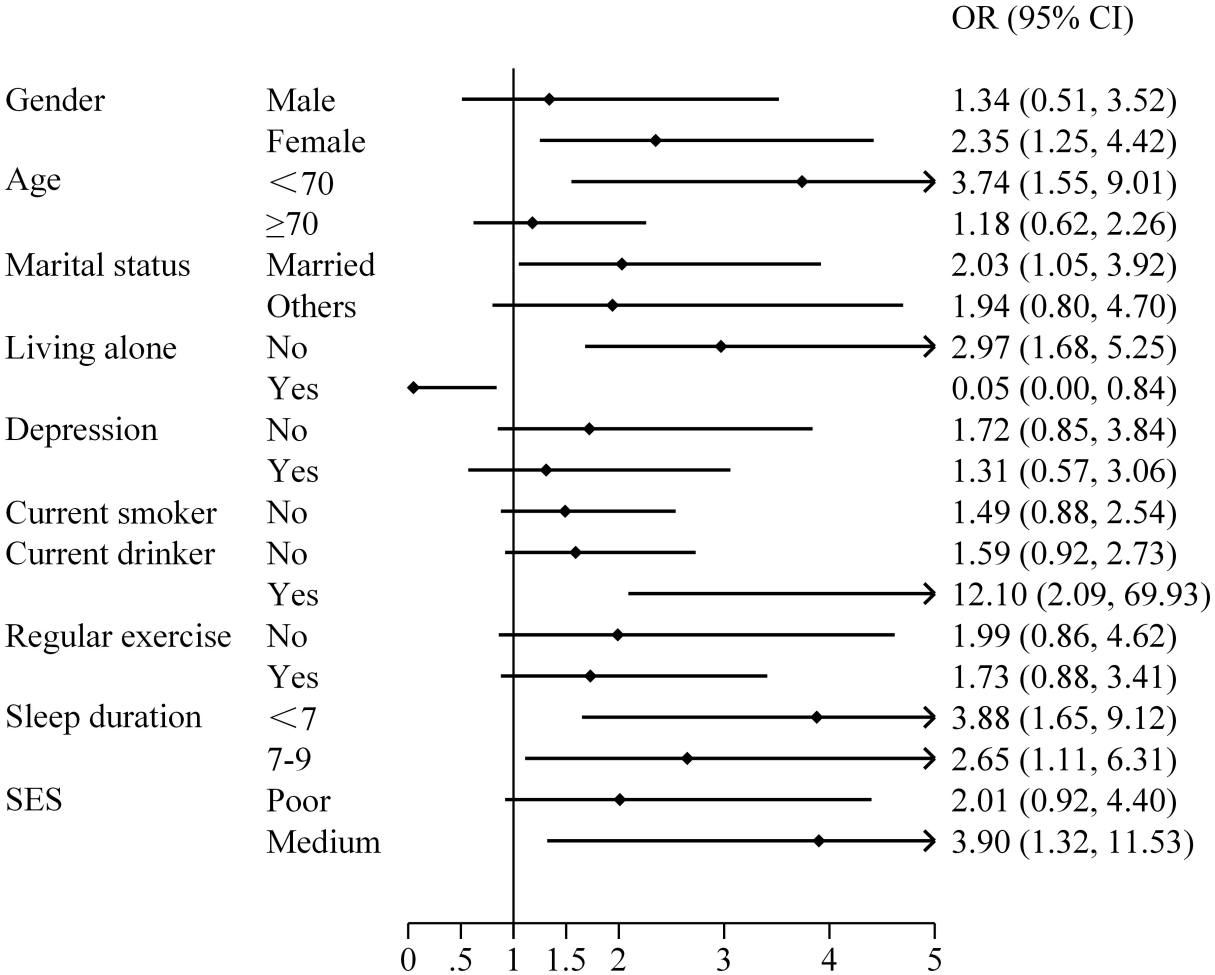
Correlation analysis between social network and cognitive impairment among different groups.

The prevalence of cognitive impairment was higher in those with poor social network (OR = 2.08, 95% CI: 1.27–3.41). Relative network 2.04 (95% CI: 1.27–3.28) and friend network 2.11 (95% CI: 1.30–3.45) were also associated with higher cognitive impairment (Table 3). We also analyzed the correlation between social networks and cognitive impairment among different groups. Male with poorer social networks had a higher risk of cognitive impairment, OR = 1.34 (95% CI: 0.51–3.52), but the results were not statistically significant, considering the lack of statistical power due to the small sample size. Similar results were found in women, OR = 2.35 (95% CI: 1.25–4.42), which also showed that the positive health effects of social networks appear to be more pronounced for women. The results for more subgroups were shown in Figure 2.

**Table 3:** Correlation analysis between social network and cognitive impairment.

	**Model 1**		**Model 2**	
	**OR (95%CI)**	***P***	**OR (95%CI)**	***P***
Social network	3.84 (2.59, 5.69)	< 0.001	2.08 (1.27, 3.41)	0.004
**Dimensions**				
Relative network	4.05 (2.75, 5.96)	< 0.001	2.04 (1.27, 3.28)	0.003
Friend network	3.57 (2.40, 5.31)	< 0.001	2.11 (1.30, 3.45)	0.003

Model 1 did not adjust for covariates, and model 2 adjusted for gender, age, marital status, night shift work, living alone, smoking status, drinking status, regular exercise, sleep duration and SES.

### 3.3. Mediated effects analysis

We further took cognitive impairment as the outcome variable, social isolation as the independent variable, depression as the mediating variable, and control related covariates to conduct the mediated effects analysis and found that the total effect (OR = 1.20, 95% CI: 1.11, 1.29) and direct effect (OR = 1.13, 95% CI: 1.04,1.22) of social network on cognitive impairment were significant. The indirect effect was statistically significant (OR = 1.06, 95% CI: 1.03, 1.10), which suggested the mediating effect of depression, and the proportion of mediating effect mediated by depression was 34.44% (Table 4).

**Table 4:** Total, direct, and indirect effects of social networks on cognitive impairment through depression.

	**OR (95% CI)**	***P***
Indirect effect	1.06 (1.03, 1.10)	< 0.001
Direct effect	1.13 (1.04, 1.22)	0.003
Total effect	1.20 (1.11, 1.29)	< 0.001
Mediated (%)	34.44	

## 4 Discussion

Although the link between social networks, depression, and cognitive impairment in older adults is well established, the exact nature of their relationship has not been conclusively determined. Using empirical data, this study found that depression has a significant mediating effect on the relationship between social isolation and cognitive impairment, suggesting that engaging in more social activity may reduce cognitive impairment by improving depressive status. This mediating effect accounts for about 1/3 of the total effect. In addition, we also found that the prevalence of cognitive impairment is high in the elderly, and social isolation increases the risk of cognitive impairment.

This study found that older adults with high social contact had better cognitive health and lower prevalence of MCI, which is consistent with many previous studies (Seeman et al., 2011; Hughes et al., 2013; Haslam et al., 2014; Glei et al., 2005; Conroy et al., 2010). This association between social networks and cognitive status can be explained by the “use it or lose it” theory. Social networks can stimulate the brain by providing social cues, organizing social gatherings, or engaging in complex social discourse, a form of mental exercise that enhances cognitive reserve and slows the process of pathophysiological changes in cognitive deterioration. There are several main hypotheses about how social networks affect cognitive state. The first is the cognitive reserve hypothesis. This hypothesis stems from the phenomenon that in some elderly populations, amyloid PET-CT molecular imaging reveals normal or only mildly impaired cognitive function despite the presence of large amounts of Aβ deposits in the brain [the main characteristic pathological change of AD is β-amyloid (Aβ) deposits]. Some scholars have therefore proposed the cognitive reserve hypothesis, which suggests that participation in social, cognitively, or intellectually stimulating activities and physical exercise activities in late middle age enhances synaptic activity and increases the capacity of the cognitive reserve, thus maintaining cognitive function at a state of MCI and delaying the clinical manifestation of dementia. The cognitive reserve hypothesis successfully explains that the nervous system can tolerate damage and pathology without manifesting clinically as functional impairment (Bennett et al., 2006). The second hypothesis is the stress hypothesis, with the observation that chronic stress increases the risk of dementia (Wilson et al., 2003), while extensive social networks may reduce stress and lower levels of hormones such as glucocorticoids and corticosteroids, which regulate brain function and slow the progression of MCI to dementia. The third hypothesis is the vascular hypothesis, which suggests that high-quality social network activity can broadly affect the biological system and reduce the risk of dementia by reducing cardiovascular disease risk factors associated with brain disease (Fratiglioni et al., 2004). In addition, a neurobiological model has been pointed out to illustrate the idea (Shen et al., 2022). Social networks can also affect cognitive function by activating neurobiological mechanisms that stimulate the hypothalamic-pituitary-adrenal axis as well as decreasing sleep quality. When a person is chronically exposed to a poor social network, he or she develops a high level of vigilance against the social environment and exhibits behaviors to stay away from it, and this self-protective escape behavior in turn deepens this prejudice against the social environment and activates neurobiological mechanisms leading to increased brain load and cognitive decline. However, findings are mixed and not all studies have reported this association (Saczynski et al., 2006; Zamora-Macorra et al., 2017; Holwerda et al., 2014). This may be due to factors such as different social environment characteristics of minority group members, differences in the composition and size of social networks, and differences in language and cultural status, which may lead to a nonsignificant relationship between social networks and cognitive impairment (Saczynski et al., 2006; Zamora-Macorra et al., 2017).

Depression is one of the most prevalent mental disorders among older adults is a global public health priority (Byers et al., 2010). Depression has a significant impact on the elderly population and is associated with higher all-cause mortality, suicide risk, and increased health service use (Manley et al., 2017). Systematic evaluations have shown that predictors of depression in older adults are cognitive impairment or less extensive social networks (Djernes, 2006). The association between social networks and depression has been widely recognized and may be a precursor to depression. Loss of interpersonal interactions in social networks, especially when combined with social exclusion, is a long recognized and valid risk factor for the development of major depression (Houtjes et al., 2014; Ford et al., 2011). A few studies have also reported no association between social networks and depression. The Descriptive Study of community-dwelling older adults by Frances et al. showed that depressed older adults were not socially isolated compared to the nondepressed group and that the depressed group reported significantly more contact with friends than the nondepressed group (Ford et al., 2011; Wilby, 2011). Two other studies also found no association between frequency of social contact and depression (Millán-Calenti et al., 2013). Depression is a well-established risk factor for MCI (Barnes et al., 2006; Teo et al., 2013). Numerous studies have confirmed that the more severe the depressive symptoms, the greater the likelihood of progression from a normal cognitive state to MCI or from MCI to dementia (Donovan et al., 2014; Rosenberg et al., 2013). Even the lowest levels of depressive symptoms induce persistent pathophysiological effects. Research by Donovan et al. (2015) pointed out that very low depressive states were an important predictor of the transition to MCI and the occurrence of neurodegenerative brain changes in a cognitively normal elderly population. Donovan et al. (2017) showed that the risk of progression from normal cognition to MCI was doubled in those with depression scores equal to or above the threshold.

The indirect association, in addition to this direct association, was found to be mediated by depressive symptoms. People with high social exposure were in better psychological condition and thus had better cognitive status. Depression mediated the association between social networks and MCI, which is consistent with the findings of several previous studies (Conroy et al., 2010; Balouch et al., 2019; Boss et al., 2015). This can be explained by a conceptual model of a social process proffered by Berkman and colleagues in the year 2000 (Fratiglioni et al., 2004). This theory suggests that extensive social contact can further influence cognitive health through mediating factors such as psychological distress. Not only does extensive social contact provide older adults with good moods, such as a sense of belonging, it can also provide timely advice and help when they experience stressful events and emergencies, which in turn reduces symptoms of depression and further hinders the progression of MCI. The study by McHugh et al. suggests that social network has an indirect effect on cognitive health through psychological stress, confirming that cognitive health mediates the association between social networks and MCI (McHugh Power et al., 2018). Demonstrating this mediation is also an important addition to further understanding of the ways in which social networks affect cognition.

There are some limitations in empirical studies. First, since the data are based on self-report, recall bias may exist. Second, because this is a cross-sectional study, the direction of the association between social networks and cognitive function cannot be determined. Third, this study used MoCA to identify cognitive impairments, which has been shown to be unstable when providing domain-specific information. Fourth, although it meets the required sample size, the small sample size may still lead to unstable research results. Fifth, the causal relationship between social networks and depression is not clear, which somewhat limits our conclusions. Sixth, we note that other social variables may be associated with cognitive health. Future research could extend the current findings by examining the role of other social factors associated with cognitive performance. Future studies in different settings are needed to validate our findings regarding the association between social networks, depression, and cognitive impairment.

## 5 Conclusion

Social isolation increases the risk of cognitive impairment and depression has a significant mediating effect on the relationship between social isolation and cognitive impairment. The discovery of the social networks-depression-cognitive impairment pathway suggests that the cognitive health of older adults can be improved by focusing on their social network status and mental health status.

## Data Availability

The raw data supporting the conclusions of this article will be made available by the authors, without undue reservation.
